# Effectiveness of adding alarms to flash glucose monitoring in adults with type 1 diabetes under routine care

**DOI:** 10.1007/s00592-022-01884-1

**Published:** 2022-04-13

**Authors:** Federico Boscari, Sara Ferretto, Francesco Cavallin, Gian Paolo Fadini, Angelo Avogaro, Daniela Bruttomesso

**Affiliations:** 1 Department of Medicine (DIMED), University of Padova, Via Giustiniani 2, 35128 Padova, Italy; 2 Division of Metabolic Diseases, University Hospital of Padova, 36020 Solagna, Italy; 3 Independent Statistician, 36020 Solagna, Italy

**Keywords:** Flash glucose monitoring-Free Style Libre 1 and 2, Type 1 diabetes, Glucose control, Time in range, Psychological aspects

## Abstract

**Aim:**

Whether glucose sensor alarms improve metabolic control and are accepted by individuals with diabetes is unclear. Here, we investigated whether switching from a standard flash glucose monitoring system (FGM1) to a system equipped with hypo- and hyperglycemia alarms (FGM2) improves glycemic control and psychological outcomes in adults with type 1 diabetes (T1D).

**Methods:**

Subjects with T1D and > 4% of time in hypoglycemia or > 40% of time in hyperglycemia were studied while wearing FGM1 (4 weeks) and after switching to FGM2 for 8 weeks. The primary endpoint was the change in time in range (TIR 70–180 mg/dl [3.9–10.0 mmol/L]) after 4 weeks of FGM2 use. Time below range (TBR), time above range (TAR), mean glucose, coefficient of variation (CV), sensor scans, treatment satisfaction, and hypoglycemia fear were secondary outcomes.

**Results:**

We included 38 subjects aged 33.7 ± 12.6 year. During 4 weeks of FGM2 use, TIR increased from 52.8 to 57.0% (*p* = 0.001), TBR decreased from 6.2 to 3.4% (*p* < 0.0001) as did time < 54 mg/dl (from 1.4 to 0.3%, *p* < 0.0001) and CV (from 39.6% to 36.1%, *p* < 0.0001). These changes were confirmed after 8 weeks of FGM2 use. Treatment satisfaction improved and fear of hypoglycemia decreased.

Subjects who had > 4% of time in hypoglycemia at baseline showed the greatest improvements in glucose control and treatment satisfaction.

**Conclusion:**

Switching from FGM1 to FGM2 improved TIR and treatment satisfaction and reduced fear of hypoglycemia. Participants who benefited most from switching from FGM1 to FGM2 were those prone to hypoglycemia.

## Introduction

Continuous glucose monitoring (CGM) measures the glucose concentration in the subcutaneous interstitial fluid every 1–5 min, informing the user about current glucose level, previous glucose trends, as well as current glucose direction and rate of change [1]. Analysis of CGM data allows estimating time in range (TIR), time above range (TAR), time below range (TBR), mean glucose, and coefficient of variation (CV) of glucose readings. This flood of information has changed how glucose control is defined and the glycemic targets individuals with diabetes are expected to meet according to their specific characteristics [2, 3].

Two types of CGM systems are now available: real-time (rt-CGM) and intermittently scanned (is-CGM, also known as flash glucose monitoring, FGM). Current rt-CGM systems send glucose values in real-time to a receiver or to a smart device, such as a watch or phone. They provide alerts and alarms for current or impending hypo- and hyperglycemia based on individualized upper and lower limits and also have alerts based on the rate of change of blood glucose [4].

FGM systems provide the same data type, but the user has to scan the sensor to obtain information. First-generation devices (FGM1) did not have alerts and alarms [5].

In people with type 1 diabetes (T1D), compared with SMBG, CGM improved glycemic control and reduced hypoglycemic episodes with greater user satisfaction [6–10]. Since their introduction, there has been a great increase in CGM use due to a progressive improvement in their accuracy and portability [11, 12]

The introduction of FGM1, easy to use and cheaper than rt-CGM, has given further impulse to the diffusion of these systems [13]. On the other hand, the lack of alarms may reduce the effectiveness of FGM1 compared to rt-CGM. Despite alarms can be perceived as invasive and generating fatigue, some studies have reported greater glycemic improvements (e.g., increased TIR, lower glycemic variability) with rt-CGM use compared with FGM1 [14–16]. A recent multicenter, randomized controlled trial [16] compared rt-CGM with FGM1 in 254 adults with type 1 diabetes (T1D), who were previously on FGM1, 17% of whom had hypoglycemia unawareness and 11% had a history of severe hypoglycemia. Six months of use of rtCGM with alert functionality improved time in range, reduced HbA1c, time with glucose < 54 mg/dL, and time in hyperglycemia [16].

Second-generation FGM (FreeStyle Libre 2, Abbott Diabetes Care, Inc., Alameda, CA, FGM2 hereafter) has become commercially available in Italy in mid-2020. Unlike FGM1, FGM2 offers optional alarms that automatically alert the user when glucose levels rise above or drop below whatever the threshold target is programmed and when the signal between sensor and reader/smartphone is lost. Unlike rt-CGM, however, no alarms are provided for impending hypo- or hyperglycemia, based on prediction algorithms.

To our knowledge, the literature offers little information on the effectiveness of FGM2 versus FGM1 in terms of glucose control. In addition, user acceptability remains a concern and is worth investigating because alarms and alerts can elicit variable responses in individuals with diabetes.

This study aimed to investigate whether switching from FGM1 to FGM2 was followed by any benefit in terms of glycemic control and psychological outcomes in adults with T1D and suboptimal glycemic control.

## Methods

### Study design

This observational study analyzed data on subjects with T1D who switched from FGM1 to FGM2 under routine clinical care. The study was performed at the Division of Metabolic Diseases of the University Hospital of Padova (Padua, Italy) following the Helsinki Declaration of 1964 and its later amendments and in agreement with national regulations. The study was conceived as a retrospective data collection and cleared by the local Ethical Committee (protocol number 151n/AO/21). All subjects provided written informed consent to use clinical data for research purposes.

### Participants

We retrospectively identified subjects aged 18 years or older, with a diagnosis of T1D from at least one year (according to World Health Organization criteria), treated with insulin pump (continuous subcutaneous insulin infusion, CSII) or multiple daily injections (MDI) who were using the FGM1 (FreeStyle Libre 1) for at least 6 months and who, over the 4 weeks preceding a regular visit, displayed suboptimal glycemic control according to the CGM consensus criteria [3]. We excluded subjects with one or more severe hypoglycemic episodes in the previous year and pregnant women.

### Data collection

Under routine clinical practice and during a scheduled consultation, selected subjects with time below range > 4% or time above range > 40% were proposed to switch to FGM2. As a working standard at our clinic, those who accepted were instructed on how to use the device and interpret alarms, set at 80 mg/dl and 200 mg/dl for low and high glucose, respectively. They also received instructions on how to react to alarms. Subjects were free to use the FGM2 reader or the smartphone app, as they did while using FGM1. No substantial change in diet or physical activity was expected during the period of FGM2 use.

During the last four weeks baseline of FGM1 use (weeks 1–4) and the first and second blocks of 4 weeks of FGM2 use (weeks 5–8 and 9–12), we calculated the following glucose metrics: time spent between 70 and 180 mg/dl (3.9–10.0 mmol/L, time in range, TIR), time below 70 or 54 mg/dl (< 3.9 and < 3 mmol/L, respectively, time below range, TBR), time between 180 and 250 mg/dl, time over 250 mg/dl (> 10.0 mmol/L and > 13.9 mmol/L, respectively) time above range, TAR), coefficient of variation of sensor glucose (CV), mean glucose, and number of scans performed with receiver/smartphone.

We compared data collected during the 4 weeks baseline (FGM1) with data gathered during weeks 5–8 on FGM2 to determine whether the switch had immediate effects. In addition, we compared data collected during weeks 9–12 of FGM2 use with data gathered during FGM1 use to evaluate if eventual immediate effects were sustained over time.

We also investigated whether the effects of switching from FGM1 to FGM2 differed between subjects who were predominantly prone to hypoglycemia or predominantly prone to hyperglycemia.

**Fig. 1:**
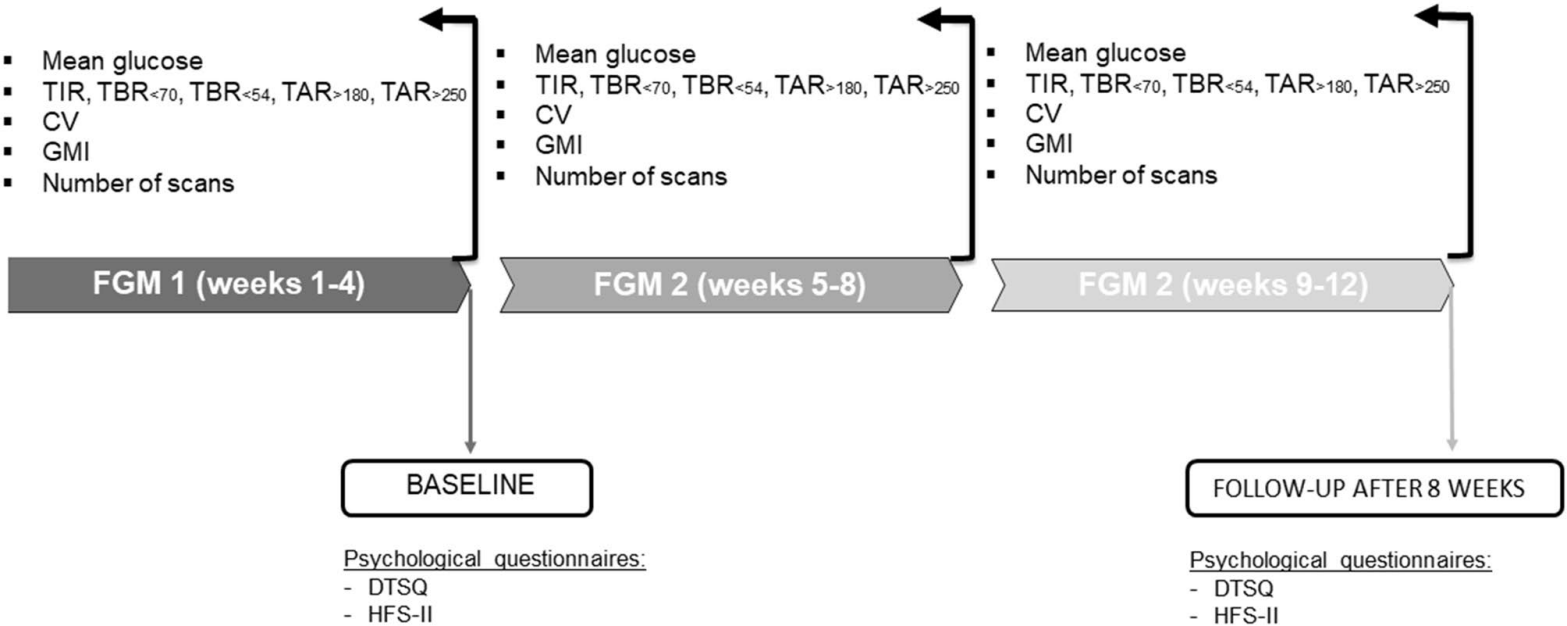
Study timeline. TIR: time in range, TBR: time below range (below 70 mg/dl and below 54 mg/dl, [< 3.9 and < 3 mmol/L, respectively]), TAR time above range (above 180 and 250 mg/dl [> 10.0 mmol/L and > 13.9 mmol/L, respectively]), CV: coefficient of variation, GMI: glucose management indicator, DTSQ: Diabetes Treatment Satisfaction Questionnaire. HFS: Hypoglycemia Fear Survey

As a standard of care at our clinic, when individuals with diabetes change medications or devices, selected subjects completed a questionnaire regarding their satisfaction about diabetes treatment (Diabetes Treatment Satisfaction Questionnaire (DTSQs), and a questionnaire regarding the fear of hypoglycemia (Hypoglycemic Fear Survey (HFS-II) immediately before switching to FGM2 and after the 8 weeks on FGM2 (Fig. 1).

DTSQs [17] consist of eight items, of which six (items 1 and 4–8) are summed to produce a measure of global treatment satisfaction, higher score suggesting higher treatment satisfaction. The remaining two items concern the perceived frequency of hyperglycemic and hypoglycemic episodes and are treated separately. (Lower scores represent lower hypo- and hyperglycemia perception.)

HFS-II [18] is a 33-item questionnaire composed of two subscales assessing behaviors (15 items) and worries (18 items) related to fear of hypoglycemia. Higher total scores indicate greater fear of hypoglycemia.

### Study outcomes

The primary outcome was the change in TIR, defined as sensor glucose of 70–180 mg/dl (3.9–10 mmol/L), after 4 weeks of FGM2 use. The main secondary outcome was a change in TIR after 8 weeks of FGM2 use. Other secondary outcomes, recorded at 4 and 8 weeks of FGM2 use, included the change in time in hypoglycemia (glucose < 70 mg/dl and < 54 mg/dl [< 3.9 and < 3 mmol/L, respectively]); time in hyperglycemia (glucose > 180 and > 250 mg/dL [> 10.0 mmol/L and > 13.9 mmol/L, respectively]); mean glucose concentration and glucose management index; coefficient of variation; number of daily sensor’s scans, fear of hypoglycemia and satisfaction for diabetes treatment.

### Statistical analysis

Continuous data were summarized as mean and standard deviation (SD) or median and interquartile range (IQR), and categorical data as frequency and percentage. Indices of glucose control were compared between FGM1 (weeks 1–4) and FGM2 (weeks 5–8 and 9–12) using paired Student t test. HFS and DRSQ were compared between FSL1 (weeks 1–4) and FSL2 (weeks 5–12) using paired Student t test (HFS total score and subscales, DTSQ total score) or Wilcoxon test (items 2 and 3 of DTSQ, which were measured on a Likert scale). Effect sizes were reported as mean differences with 95% confidence interval (CI), or median of the differences with bootstrap 95% CI. All tests were two-sided, and a p value less than 0.05 was considered significant. Statistical analysis was performed using R 4.1 (R Foundation for Statistical Computing, Vienna, Austria) [19].

### Results

We identified 38 participants (17 men and 21 women; mean age 33.7 years) with T1D who had suboptimal glycemic control (mean HbA1c 7.7%, 61 mmol/mol), 68.4% of whom was treated with MDI (Table 1).

**Table 1:** Subjects characteristics

N subjects	38
Age, years ^a^	33.7 (12.6)
Males	17 (44.7%)
BMI, kg/m^2 a^	24.6 (5.3)
Duration of diabetes, years ^a^	21.2 (12.0)
HbA1c, %^a^	7.7 (1.0)
HbA1c, mmol/mol ^a^	60.9(10.7)
Insulin Therapy:	
MDI ^a^	26 (68.4%)
CSII ^a^	12 (31.6%)
Insulin requirement, IU/kg/day	
Total bolus insulin ^a^	0.27 (0.10)
Total basal insulin ^a^	0.32 (0.11)
Subjects with 1 or more chronic complications	12 (31.6%)
Subjects with TBR > 4% using FGM1	19 (50%)
Subjects with TAR > 40% using FGM1	15 (39%)
Subjects with time in TBR > 4% and TAR > 40% using FGM1	4 (11%)

Data expressed as *n* (%) or ^a^ mean (SD)

While participants were using FGM1 (weeks 1–4), mean TIR was 52.8% (SD 13.6), mean TBR was 6.2% (SD 4.9) with 1.4% (SD 1.6) of time spent below 54 mg/dL. TAR was 41.1% (SD 17.1) with 14.6% (SD 11.5) of time spent above 250 mg/dl. After 4 weeks of FGM2 use, there was a significant improvement of time in range (from 52.8 to 57.0%, *p* = 0.001), a marked reduction of the time spent < 70 mg/dl (< 3.9 mmol/), from 6.2 to 3.4% (*p* < 0.0001), and an even greater reduction of the time spent below 54 mg/d (< 3 mmol/l), from 1.4 to 0.3% (*p* < 0.0001), which was fourfold lower while on FGM2. No changes were observed in mean glucose, but there was a significant reduction of CV from 39.6 to 36.1%, *p* < 0.0001. While wearing FGM2, participants increased scanning from 9.6 to 15.5 times per day (*p* < 0.0001).

Remarkably, these changes, apart from the time spent > 250 mg/dl, were confirmed after 8 weeks of FGM2 use (Table 2).

**Table 2:** Glucose control: comparison between FGM1 (weeks 1–4) and FGM2 periods (weeks 5–8 and 9–12)

Outcome measure	FGM1 (weeks 1–4)	FGM2 (weeks 5–8)	FGM2 (weeks 5–8) versus FGM1 (weeks 1–4)		FGM2 (weeks 9–12)	FGM2 (weeks 9–12) versus FGM1 (weeks 1–4)	
	Mean (SD)	Mean (SD)	MD (95% CI)	*p* value	Mean (SD)	MD (95% CI)	*p* value
Time in 70–180 mg/dl, %	52.8 (13.6)	57.0 (14.3)	4.2 (1.8 to 6.7)	0.001	55.8 (15.9)	3.0 (0.1 to 5.8)	0.04
Time below 70 mg/dl, %	6.2 (4.9)	3.4 (3.0)	− 2.8 (− 3.7 to − 1.7)	< 0.0001	3.6 (3.3)	− 2.6 (− 3.6 to − 1.5)	< 0.0001
Time below 54 mg/dl, %	1.4 (1.6)	0.3 (0.7)	− 1.1 (− 1.6 to − 0.6)	< 0.0001	0.5 (1.0)	− 0.9 (− 1.4 to − 0.4)	0.0005
Time above 180 mg/dl, %	41.1 (17.1)	39.6 (15.4)	− 1.5 (− 4.3 to 1.3)	0.27	40.6 (17.6)	− 0.5 (− 3.8 to 2.8)	0.77
Time above 250 mg/dl, %	14.6 (11.5)	11.9 (9.4)	− 2.7 (− 4.9 to − 0.5)	0.02	13.6 (11.6)	− 1.1 (− 3.6 to 1.3)	0.35
Mean glucose, mg/dl	171.2 (32.1)	169.6 (24.2)	− 1.6 (− 6.9 to 3.7)	0.54	171.6 (29.7)	0.4 (− 5.5 to 6.3)	0.89
GMI, %	7.4 (0.7)	7.4 (0.6)	0.0 (− 0.2 to 0.1)	0.54	7.4 (0.7)	0.0 (− 0.1 to 0.1)	0.89
CV, %	39.6 (5.3)	36.1 (5.5)	− 3.5 (− 4.9 to − 2.1)	< 0.0001	36.3 (4.7)	− 3.3 (− 4.8 to − 1.8)	< 0.0001
Number of scans per day	9.6 (6.5)	15.5 (7.8)	5.9 (4.3 to 7.5)	< 0.0001	13.4 (7.1)	3.8 (2.4 to 5.2)	< 0.0001

*CI* confidence interval. *CV* coefficient of variation, *GMI* glucose management indicator, *MD* mean difference, *SD* standard deviation

In 19 subjects with TBR > 4% while on FGM1, who had a mean (SD) HbA1c of 7.0 (0.7) % (53.4 (8.0) mmol/mol), switching to FGM2 increased TIR and number of scans per day and decreased TBR and CV (Table 3). In 15 subjects with TAR > 40% on FGM1 who had a mean (SD) HbA1c of 8.5% (0.8) (69.0 (8.3) mmol/mol), switching to FGM2 increased number of scans per day and slightly decreased TBR (< 54 mg/dl), with no effect on TAR (Table 3).

**Table 3:** Glucose control: comparison between FGM1 (weeks 1–4) and FGM2 (weeks 5–12) in the subgroup of subjects with TBR > 4% and the subgroup of subjects with TAR > 40% at baseline with FSL1

	Subjects with TBR > 4% (*n* = 19)				Subjects with TAR > 40% (*n* = 15)			
Outcome measure	FGM1 (weeks 1–4)	FGM2 (weeks 5–12)	FGM2 (weeks 5–12) versus FGM1 (weeks 1–4)		FGM1 (weeks 1–4)	FGM2 (weeks 5–12)	FGM2 (weeks 5–12) vs. FGM1 (weeks 1–4)	
	Mean (SD)	Mean (SD)	MD (95% CI)	*p* value	Mean (SD)	Mean (SD)	MD (95% CI)	*p* value
Time in 70–180 mg/dl, %	63.6 (8.8)	66.9 (10.3)	3.3 (0.7 to 5.9)	0.02	41.0 (7.9)	44.1 (11.6)	3.1 (− 1.8 to 8.0)	0.20
Time below 70 mg/dl, %	9.7 (4.1)	5.1 (3.2)	–4.5 (–5.9 to –3.2)	< 0.0001	1.6 (1.2)	1.5 (1.4)	− 0.1 (–0.6 to 0.3)	0.54
Time below 54 mg/dl, %	2.2 (1.7)	0.6 (0.9)	− 1.6 (− 2.4 to − 0.7)	0.001	0.3 (0.4)	0.0 (0.0)	− 0.3 (− 0.5 to − 0.1)	0.04
Time above 180 mg/dl, %	26.8 (9.2)	28.0 (9.8)	1.2 (–1.8 to 4.2)	0.41	57.5 (8.4)	51.3 (11.3)	− 6.2 (− 14.1 to 1.7)	0.11
Time above 250 mg/dl, %	6.0 (4.4)	5.3 (3.9)	− 0.7 (-2.4 to 1.1)	0.43	24.3 (9.9)	19.2 (8.6)	− 5.1 (− 11.7 to 1.6)	0.12
Mean glucose, mg/dl	145.1 (15,4)	150.4 (13.9)	5.2 (0.0 to 20.4)	0.05	201.3 (20.5)	192.0 (16.7)	− 9.2/ − 20.4 to 1.9)	0.10
GMI, %	6.8 (0.4)	6.9 (0.3)	0.1 (0.0 to 0.2)	0.05	8.1 (0.5)	7.9 (0.4)	− 0.2 (− 0.5 to 0.1)	0.10
CV, %	41.7 (4.5)	36.6 (4.8)	− 5.1 (− 6.8 to − 3.3)	< 0.0001	36.0 (4.7)	35.4 (5.0)	0.6 (− 2.5 to 1.3)	0.51
Number of scans per day	9.6 (5.4)	16.0 (7.4)	6.4 (4.1 to 8.8)	< 0.0001	9.9 (8.5)	13.0 (7.8)	3.1 (1.5 to 4.6)	0.0007

C*I* confidence interval, *CV* coefficient of variation, *GMI* glucose management indicator, *MD*, mean difference, *SD*, standard deviation

Compared to the period of FGM1 use, during FGM2 use, there was a greater treatment satisfaction. Using the DTSQs, the mean global score increased from 26.7 to 29.8 (*p* = 0.004). No differences were observed in questions 2 and 3, regarding the perception of hyper- or hypoglycemia (Table 4). Results obtained using the HFS-II questionnaire demonstrated a decreased fear of hypoglycemia, reducing HFSII scores globally and in its subscales (Table 4).

**Table 4:** Treatment satisfaction and perception of hyper/hypoglycemia (as measured by DTSQ) and fear of hypoglycemia (as measured by HFS): comparison between FGM1 (weeks 1–4) and FGM2 (weeks 5–12)

Outcome measure	FGM1 (weeks 1–4)	FGM2 (weeks 5–12)	FGM2 (weeks 5–12) versus FGM1 (weeks 1–4)	
	Mean (SD)	Mean (SD)	MD (95% CI)	*p* value
DTSQ: global satisfaction	26.7 (5.4)	29.6 (5.9)	2.2 (0.6 to 3.7)	0.007
	Median (IQR)	Median (IQR)	Median of the differences (95% CI)	*p* value
DTSQ: question concerning perception of hyperglycemia	4 (3–5)	3 (2–3)	− 1 (− 2 to 0)	0.37
DTSQ: question concerning perception of hypoglycemia	3 (2–4)	3 (2–3)	0 (− 1 to 0)	0.99
	Mean (SD)	Mean (SD)	MD (95% CI)	p value
HFS: total	43.8 (25.9)	28.6 (19.4)	− 15.2 (− 25.3 to − 5.1)	0.004
HFS: behaviors	20.8 (10.2)	14.9 (9.5)	− 5.9 (− 10.5 to − 1.4)	0.01
HFS: worries	22.9 (16.6	13.7 (11.5)	− 9.3 (− 15.3 to − 3.3)	0.003

*CI* confidence interval, *DTSQ* Diabetes Treatment Satisfaction Questionnaire, *HFS* Hypoglycemia Fear Survey, *MD* mean difference, *SD* standard deviation

Similarly, a decrease in fear of hypoglycemia was observed in 19 subjects with TBR > 4% and 15 subjects with TAR > 40% at baseline with FGM1, while improved satisfaction about treatment was observed only in 19 subjects with TBR > 4% (Table 5).

**Table 5:** Treatment satisfaction and perception of hyper-/hypoglycemia (as measured by DTSQ) and fear of hypoglycemia (as measured by HFS): comparison between FGM1 (weeks 1–4) and FGM2 (weeks 5–12) in the subgroup of subjects with TBR > 4% and the subgroup of subjects with TAR > 40% at baseline with FGM1

	Subjects with TBR > 4% (*n* = 19)				Subjects with TAR > 40% (*n* = 15)			
Outcome measure	FGM1 (weeks 1–4)	FGM2 (weeks 5–12)	FGM2 (weeks 5–12) versus FGM1 (weeks 1–4)		FGM1 (weeks 1–4)	FGM2 (weeks 5–12)	FGM2 (weeks 5–12) versus FGM1 (weeks 1–4)	
	Mean (SD)	Mean (SD)	MD (95% CI)	*p* value	Mean (SD)	Mean (SD)	MD (95% CI)	*p* value
DTSQ: global satisfaction	26.6 (5.3)	31.7 (5.0)	3.5 (1.2 to 5.8)	0.006	26.9 (5.5)	27.6 (6.0)	0.8 (− 1.5 to 3.2)	0.45
	Median (IQR)	Median (IQR)	Median of the differences (95% CI)	*p* value	Median (IQR)	Median (IQR)	Median of the differences (95% CI)	*p* value
DTSQ: question concerning perception of hyperglycemia	3 (3–4)	3 (1–3)	− 1 (− 2 to 0)	0.99	5 (4–5)	3 (3–4)	− 1 (− 2 to 0)	0.39
DTSQ: question concerning perception of hypoglycemia	3 (3–5)	3 (2–3)	− 1 (− 1 to 0)	0.99	2 (1–2)	2 (1–3)	0 − 1 to 1)	0.99
	Mean (SD)	Mean (SD)	MD (95% CI)	*p* value	Mean (SD)	Mean (SD)	MD (95% CI)	*p* value
HFS: total	44.5 (30.3)	26.5 (19.6)	− 17.9 (− 35.5 to − 0.3)	0.04	42.6 (20.6)	28.7 (20.3)	− 13.9 (− 27.2 to − 0.6)	0.04
HFS: behaviors	21.2 (11.4)	13.7 (8.5)	− 7.5 (− 14.6 to − 0.3)	0.04	20.5 (9.3)	14.2 (10.2)	− 6.3 (− 13.6 to 1.0)	0.09
HFS: worries	23.3 (19.4)	12.8 (12.7)	− 10.5 (− 21.3 to 0.3)	0.06	22.1 (12.7)	14.4 (11.2)	− 7.7 (− 14.8 to − 0.6)	0.04

*CI* confidence interval,* DTSQ* Diabetes Treatment Satisfaction Questionnaire, *HFS*, Hypoglycemia Fear Survey, *MD*, mean difference, *SD* standard deviation

## Discussion

This study shows that adults with T1D and insufficient control of hyper- or hypoglycemia while using FGM1 exhibited significant glycemic and psychological improvements after switching to FGM2. To our knowledge, there is only one prior study evaluating FGM2 in subjects with T1D, but differently to our study, such comparison concerned a pediatric population, and FGM2 was compared to SMBG [20].

We found that FGM2 increased the time in range and reduced the time spent in hypoglycemia. Interestingly, the time spent in hypoglycemia was significantly decreased after the first 4 weeks of FGM2 use, suggesting that FGM2 is easy to use and quickly advantageous over FGM1. Reassuringly, effectiveness in terms of hypoglycemia reduction was maintained during the subsequent 4 weeks to a total of 8 weeks.

Despite the reduction in hypoglycemia there was a modest, non-significant increase in mean glucose and GMI. GMI reflects average glucose levels and the small change observed in this study is due to the contribution of glucose readings during the TBR (which decreased significantly from 6.2 to 3.6%), to the greater number of readings during TAR (which decreased non-significantly from 41.1 to 40.6% of total time) and to readings during the TIR (which increased significantly from 52.2 to 55.8%).

FGM2 was not as strongly effective in reducing the time in hyperglycemia., which was significantly improved only during the first 4 weeks of FGM2 use and just for values above 250 mg/dl. The lack of significance of hyperglycemia reduction over the entire 8 weeks of the study (*p* = 0.07) may have different explanations. On one side, the hyperglycemia threshold set at 200 mg/dL, when time above range was > 40% could have caused too many alarms leading subjects to silence or ignore them. On the other side, our education programs might be more focused on avoiding hypoglycemia than correcting hyperglycemia. Of note, although threshold choice is considered important [21], there are no formal guidelines for selecting thresholds for hypoglycemia and hyperglycemia alerts. Some have suggested a starting threshold of 70 mg/dL for hypoglycemia and 250 mg/dL for hyperglycemia [22].

The increase in time in range was statistically significant but small, such that it remained well below the recommended percentage [3]. We argue that a further educational support could have improved these results. It is clear, however, that subjects not reaching the recommended targets while using FGM2, even with improved education, should be offered more advanced technologies.

In our study, the use of FGM2 was associated with an increase in the number of scans, likely due to a reaction to alarms. This increase in scan frequency can positively affect glucose control, as shown in real-life studies on FGM1 efficacy [23]. The presence of alarms did not worsen diabetes treatment satisfaction, but rather it improved satisfaction in subjects spending more time in hypoglycemia at baseline, as indicated by the fact that the improvement in DTSQ score was limited to this group of participants.

Hypoglycemia reduction was associated with a decreased fear of hypoglycemia, as demonstrated by HFS-II results. Fewer worries related to hypoglycemia and changes in behaviors adopted to avoid hypoglycemia, which may be due to alarms, were obtained despite the relatively short duration of observation, implying that the population who benefits most from FGM2 is made by subjects prone to hypoglycemia. People with a baseline time in hypoglycemia > 4% had the most significant improvement in glucose control after switching to FGM2. We acknowledge, however, that the small sample size suggests caution in the interpretation of results.

This study has some limitations. First, it was performed at a single center, with long-standing experience in T1D management, such that generalizability to less specialized clinical care centers is unclear. Second, this study had a relatively short duration, and results should be confirmed with more extended observation. Third, FGM2 use was evaluated in people with T1D and poor glucose control. Thus, data regarding hypoglycemia reduction should be confirmed in subjects at lower risk of hypoglycemia. On the other hand, it appears that those who should benefit most from alarms are those at greatest risk of hypoglycemia.

Of note, even if MARD of FGM1 and FGM2 is 9.2%, as declared by Abbott, both devices are less accurate during hypoglycemia and may fail to detect all hypoglycemic events.

Beyond these limits, our study reported for the first time data regarding effectiveness and acceptance of switching from FGM1 to FGM2 among adults with T1D.
